# Association between Blood Dioxin Level and Chronic Kidney Disease in an Endemic Area of Exposure

**DOI:** 10.1371/journal.pone.0150248

**Published:** 2016-03-10

**Authors:** Chien-Yuan Huang, Cheng-Long Wu, Jin-Shang Wu, Jung-Wei Chang, Ya-Yun Cheng, Yau-Chang Kuo, Yi-Ching Yang, Ching-Chang Lee, How-Ran Guo

**Affiliations:** 1 Department of Environmental and Occupational Health, College of Medicine, National Cheng Kung University, Tainan, Taiwan; 2 Tainan Science Park Clinic, Chi-Mei Medical Center, Tainan, Taiwan; 3 Department of Occupational Safety and Health, Chang Jung Christian University, Tainan, Taiwan; 4 Department of Occupational and Environmental Medicine, National Cheng Kung University Hospital, Tainan, Taiwan; 5 Department of Occupational and Environmental Medicine, College of Medicine, National Cheng Kung University, Tainan, Taiwan; 6 Department of Family Medicine, College of Medicine, National Cheng Kung University, Tainan, Taiwan; 7 Research Center for Environmental Trace Toxic Substances, National Cheng Kung University, Tainan, Taiwan; 8 Occupational Safety, Health, and Medicine Research Center, National Cheng Kung University, Tainan, Taiwan; School of Public Health of University of São Paulo, BRAZIL

## Abstract

**Background:**

Dioxin is an industrial pollutant related to various diseases, but epidemiological data on its effects on the kidney are limited. Therefore, we conducted a study to evaluate the association between dioxin exposure and chronic kidney disease (CKD) and identify the related factors.

**Methods:**

We conducted a community-based cross-sectional study and recruited participants from an area where the residents were exposed to dioxin released from a factory. We defined a “high dioxin level” as polychlorinated dibenzo-p-dioxins and dibenzofurans (PCDD/Fs) ≥ 20 pg WHO_98_-TEQ_DF_/g lipid in the serum and defined CKD as having an estimated glomerular filtration rate (e-GFR) ≤ 60 mL/min/1.73m^2^ or a diagnosis of CKD by a physician. The renal function was assessed between 2005 and 2010, and we excluded those who had had kidney diseases before the study started. Comparisons between patients of CKD and those who did not have CKD were made to identify the risk factors for CKD.

**Results:**

Of the 2898 participants, 1427 had high dioxin levels, and 156 had CKD. In the univariate analyses, CKD was associated with high dioxin levels, age, gender, metabolic syndrome, diabetes mellitus, hypertension, and high insulin and uric acid levels. After adjusting for other factors, we found high dioxin levels (adjusted odds ratio [AOR] = 1.76, 95% confidence interval [CI]: 1.04–2.99), female gender (AOR = 1.74, 95%CI: 1.20–2.53), hypertension (AOR = 1.68, 95%CI: 1.17–2.42), high insulin levels (AOR = 2.14, 95% CI: 1.26–3.61), high uric acid levels (AOR = 4.25, 95% CI: 2.92–6.20), and older age (AOR = 4.66, 95% CI: 1.87–11.62 for 40–64 year and AOR = 26.66, 95% CI: 10.51–67.62 for age ≥ 65 year) were independent predictors of CKD.

**Conclusion:**

A high dioxin level was associated with an increased prevalence of CKD. Therefore, the kidney function of populations with exposure to dioxin should be monitored.

## Introduction

With the economical development, more and more industrial pollutants are released to the environment, and many of them become threats to the human health. Among those, polychlorinated dibenzo-p-dioxins and dibenzofurans (PCDD/Fs) are well-known for their health effects. After entering the human body, dioxin compounds will accumulate and may cause various adverse health effects [1]. At high doses, such effects may include body weight loss, growth retardation, thymic atrophy, subcutaneous edema, and degeneration of the cardiovascular system; adverse effects have been also noted on the liver, gastro-intestinal tract, urogenital tract, and skin [2,3]. In low-dose animal experiments, indirect damage to cells and suppression of the humoral immunity were observed in mice, and the bodily load was found to be the important attribute of the toxic effects [2–4]. In addition, tetrachlorodibenzodioxin (TCDD), the most potent congener of PCDD/Fs, can affect the reproduction system of fish, birds, and mammals. It can decrease the weights of organs such as testicles and reduce seminal fluid production, reproductive capacity, testosterone synthesis, serum testosterone density, and pituitary secretion of luteinizing hormone [2,5]. It can also lead to ovary dysfunction, reduction of the reproductive capacity in the feminine part, difficulties in conception, endometrium dysplasia, and changes in the ovary hormone density, the size and weight of the uterus, the menstruation, and the estrogen circulation [6,7].

It is generally believed that most toxic effects of dioxins and dioxin-like polychlorinated biphenyls (PCB) congeners are mainly mediated by the aryl hydrocarbon receptor (AhR) [8]. The interaction of TCDD with the AhR can induce the expression of renal cytochrome P450 (CYP)-A1 and CYP-B1 proteins [9], which contribute significantly to the induction of reactive oxygen species (ROS) formation and cause renal toxicity [10,11]. The activation of AhR by TCDD may also induce inflammatory responses of kidney tubular cells, and inflammation plays an important role in the pathophysiology of chronic kidney disease (CKD) [12]. It is also possible that dioxin may affect the renal function through indirect effects [13]. In fact, an animal study found a negative correlation between the 2,3,7,8-TCDD concentration and the glomerular filtration rate (GFR), and the impairment of renal function was related to the toxicity of 2,3,7,8-TCDD [14]. In addition, combined exposure to TCDD and PCBs were found to induce significant renal toxicity in rats, and there were complicated interactions between the two pollutants [15].

While animal experiments have demonstrated the biological plausibility of the renal toxicity of dioxin, most previous studies on PCDD/Fs were focused on 2,3,7,8-TCDD, and few epidemiology studies have been conducted on the renal toxicity of dioxin on human. Therefore, we conducted a study to evaluate the association between dioxin and CKD and the related factors.

## Materials and Methods

### Study Population

We conducted a community-based cross-sectional study in the An-nan District of the Tainan City in southwestern Taiwan, where a factory produced PCP daily between 1965 and 1979. PCDD/Fs were byproducts of the manufacturing process, and a lot of them were left at the original location after the shutdown of the factory in 1982. The factory is less than 2 km from a major residential area and is at the west end of the Tainan City, near the coast. The chemicals were washout by the rainfall to the sea reservoir, and a study found that dioxin contents of the sediment reached as high as 1000 to 6000 pg-TEQ/g dry weight [16]. A series of studies were carried out, and residents living in the vicinity of the deserted factory were found to have high daily intakes of PCDD/Fs from food, especially seafood, and high PCDD/Fs levels in the serum [17,18]. In a previous survey, the average serum PCDD/Fs level of the residents in this area was 53.4 pg WHO_98_-TEQ_DF_/g lipid, while levels in other parts of Taiwan typically ranged from 15 to 20 pg WHO_98_-TEQ_DF_/g lipid [17]. During the operation of the factory, mercury-contaminated sludge and water were also dumped into the Luermen River nearby, and some of the residents ate the fish caught from the river. As a result, high serum mercury levels were also found in some of the residents [18].

The whole exposure area covers 3 of the 51 administrative subdivisions (called “Li” in Taiwan) of the An-nan District: Hsien-Gong, Lu-Erh, and Ssu-Tsao. As previous studies showed that the average serum PCDD/Fs level in the general population of Taiwan typically ranging from 15 to 20 pg WHO_98_-TEQ_DF_/g lipid [19], we define a “high dioxin level” as a serum PCDD/Fs ≥ 20pg WHO_98_-TEQ_DF_/g lipid. While we also made comparisons among groups defined by quartiles, we focused on comparing between participants with and without a high level in further analyses. We adopted the serum levels measured by the Bureau of Health of the Tainan City using isotope dilution high-resolution gas chromatography/high-resolution mass spectrometry between 2005 and 2007; detailed methodology of the measurements has been described in previous reports [17,18,20]. We invited all residents in the exposure area above 18 years of age to participate in this study. Those who had congenital kidney disease, IgA nephropathy, post infectious kidney disease, or medicine induced kidney disease were excluded from the study population.

### Assessment of Health Outcomes

We performed health examinations on each participant from July 2005 to May 2010. According to the results, we calculated the estimated GFR (e-GFR) and defined CKD as having an e-GFR ≤ 60 mL/min/1.73m^2^ or a diagnosis of CKD by a physician on the basis of the K/DOQI clinical practice guidelines [21]. In other words, we were interested in CKD stage III or higher. In calculating the e-GFR, we used the simplified equation developed by the Modification of Diet in Renal Disease (MDRD) Study [22], which is generally adopted by clinicians in Taiwan. The creatinine level was measured using the Jaffe kinetic assay.

Using results of the health examination of each participant, we also evaluate the existence of metabolic syndrome (MS), which was defined as meeting three of the following criteria: fasting glucose ≥ 100 mg/dL or under diabetes medication, waist circumference ≥ 90 cm in men or ≥ 80 cm in women, triglycerides ≥ 150 mg/dL or under drug treatment for elevated triglycerides, high-density lipoprotein (HDL) < 40 mg/dL in men or < 50 mg/dL in women, and blood pressures ≥ 130/85 mmHg or under antihypertensive medication. The criteria are implemented by the Taiwan government in concordance with the newly developed harmonized definition of MS [23,24], including the use of “population- and country-specific definitions” for “elevated waist circumference,” i.e. ≥ 90 cm in men or ≥ 80 cm in women. The definition has been adopted by many studies, including a previous study of our team [25]. We defined diabetes mellitus (DM) as having a fasting glucose > 126 mg/dL or under diabetes medication.

To control effects of potential confounders, we used a self-administrated questionnaire to collect data on demographic characteristics and medical history at the same time when the health examination was performed.

### Data Analysis

To identify the risk factors for CKD, we compared the prevalence of each potential risk factor between participants with and without CKD and evaluated the differences using chi-square tests. We adopted the normal ranges in our laboratory to determine the cutoffs, which were 22.0 mU/L for insulin, 7 mg/dl for uric acid, and 100 mg/dl for fasting glucose. The cutoffs for age were determined according to the definition of elderly (65 years old) and the age for implementing periodical health examination (40 years old) by the Taiwan government. The cutoff value for blood mercury level was determined according to a previous survey of a representative sample of the whole Taiwan area [26]. We also divided the participants by quartiles of PCDD/Fs and evaluated the differences in eGFR among the groups by analysis of variance (ANOVA).

To evaluate the effects of potential risk factors, we performed univariate logistic regressions and calculated the odds ratio (OR) and associated 95% confidence interval (CI) for each variable. Furthermore, we performed multiple logistic regressions to identify independent predictors of CKD and evaluate their effects. We constructed a “full model” in the initial analysis which included all potential risk factors and a “final model” in the further analysis which included predictors with statistical significance only. In both analyses, an adjusted odd ratio (AOR) and the associated 95% CI were calculated for each potential risk factor. In building the “final model,” we applied a stepwise approach which removed one variable in one step. We checked the variance inflation factor and evaluated the goodness of fit of the final model using the Hosmer-Lemeshow test.

We analyzed the data using SPSS Version 15.0. All statistical tests were performed at the two-tailed significance level of 0.05.

### Ethics Statement

The protocol of this study was approved by the Institution Review Board of the National Cheng Kung University Hospital. All the participants provided their written informed consent to participate in this study, and the inform consent form had been approved by the Institution Review Board of the National Cheng Kung University Hospital.

## Results

Of the residents above 18 years old in the exposure area, 3034 (85%) participated in this study. We excluded 124 with incomplete data and 12 with congenital kidney disease, IgA nephropathy, post infectious kidney disease, or medicine induced kidney disease. The serum levels of PCDD/Fs had a median of 19.60 pg WHO_98_-TEQ_DF_/g lipid and an inter-quartile range of 11.30 to 35.33 pg WHO_98_-TEQ_DF_/g lipid. Of the 2898 qualified candidates to participate in the study, 1427 had high dioxin levels, and 180 had CKD, yielding a CKD prevalence of 6.2%. In comparison with participants without high dioxin levels, those with high dioxin were older and had higher prevalence of female gender, MS, DM, hypertension, high insulin (≥ 22 mU/L), and high uric acid (> 7 mg/dL). (Table 1)

**Table 1:** Distributions (number and percentage) of variables in the participants and comparisons of variables between participants with and without a high level of dioxin.

	Total	PCDD/Fs (pg WHO98-TEQDF/g lipid)		
Variables		< 20	≥ 20	p^e^
	N (%)^d^	N (%)	N (%)	
Gender				< 0.001
Men	1546 (53.3)	870 (56.3)	676 (43.7)	
Women	1352 (46.7)	601 (44.5)	751 (55.5)	
Age (year)				< 0.001
< 40	1124 (38.8)	972 (86.5)	152 (13.5)	
40–64	1188 (41.0)	429 (36.1)	759 (63.9)	
≥ 65	586 (20.2)	70 (11.9)	516 (88.1)	
Metabolic syndrome^a^				< 0.001
No	1973 (71.5)	1179 (59.8)	794 (40.2)	
Yes	785 (28.5)	273 (34.8)	512 (65.2)	
Diabetes mellitus^b^				< 0.001
No	2472 (85.3)	1384 (56.0)	1088 (44.0)	
Yes	426 (14.7)	88 (20.2)	339 (79.8)	
Hypertension				<0.001
No	2411 (83.3)	1373 (56.9)	1038 (43.1)	
Yes	485 (16.7)	96 (19.8)	389 (80.2)	
Insulin (mU/L)				0.003
< 22.0	2579 (93.3)	1375 (53.3)	1204 (46.7)	
≥ 22.0	186 (6.7)	78 (41.9)	108 (58.1)	
Uric acid (mg/dL)				0.025
≤ 7	2276 (78.5)	1180 (51.8)	1096 (48.2)	
> 7	622 (21.5)	291 (46.8)	331 (53.2)	
Chronic kidney disease^c^				< 0.001
No	2718 (93.8)	1447 (53.2)	1271 (46.8)	
Yes	180 (6.2)	24 (13.3)	156 (86.7)	
Mercury (ppb)				<0.001
≤ 20	2687 (92.7)	1416 (52.7)	1271 (47.3)	
> 20	211 (7.3)	55 (26.1)	156 (73.9)	

^a^defined as meeting three of the following criteria: fasting glucose ≥ 100 mg/dL or under diabetes medication, waist circumference ≥ 90 cm in men or ≥ 80cm in women, triglycerides ≥ 150 mg/dL or under drug treatment for elevated triglycerides, high-density lipoprotein (HDL) < 40 mg/dL in men or < 50 mg/dL in women, and blood pressures ≥ 130/85 mmHg or under antihypertensive medication; data not available on 140 participants without high exposure

^b^defined as fasting glucose > 126 mg/dL or under diabetes medication

^c^defined as having an estimated glomerular filtration rate ≤ 60 mL/min/1.73m^2^, an e-GFR > 60 ml/min/1.73m^2^ with proteinuria, or a previous diagnosis of chronic kidney disease by a physician

^d^column percentage

^e^for chi-square test

When we divided the participants by quartiles of PCDD/Fs, we observed that as PCDD / Fs increased, the eGFR decreased, indicating a negative association between the PCDD/Fs level and e-GFR (p < 0.001 for ANOVA). (Table 2) Positive associations were observed between the PCDD/Fs level and the mercury level, age, fasting glucose, and insulin level (all with p < 0.001).

**Table 2:** Means and standard errors (SEs) of variables according to quartiles of PCDD/Fs.

Variables	Quartiles of PCDD/Fs^a^				
Mean (SE)	Q1	Q2	Q3	Q4	p^b^
Mercury (ppb)	7.14 (0.17)	9.76 (0.25)	11.83 (0.32)	12.05 (0.32)	<0.001
Age (year)	31.02 (0.38)	41.90 (0.51)	53.78 (0.54)	62.16 (0.52)	<0.001
Fasting glucose (mg/dL)	91.25 (0.69)	96.72 (1.23)	106.39 (1.50)	110.90 (1.52)	<0.001
Insulin (mU/L)	8.70 (0.35)	8.16 (0.34)	9.75 (0.40)	11.27 (0.77)	<0.001
Uric acid (mg/dL)	5.75 (0.06)	5.78 (0.09)	5.90 (0.06)	5.93 (0.09)	0.210
eGFR (mL/min/1.73m^2^)	109.08 (0.91)	102.07 (0.97)	91.91 (0.93)	87.71 (1.13)	<0.001

^a^the cut-offs are 11.30, 19.60, and 35.33 pg WHO_98_-TEQ_DF_/g lipid

^b^for ANOVA

Using 20 pg WHO_98_-TEQ_DF_/g lipid as the cutoff, we found the prevalence of CKD was higher in participants with high dioxin levels (1.6% vs. 10.9%, p < 0.001). (Table 3) The risk of CKD was higher in participants who were older or female, had high levels of dioxin, insulin, or uric acid, or had DM, hypertension, or MS (all with p < 0.05). (Table 3)

**Table 3:** Comparisons between participants with and without chronic kidney disease (CKD).

	Without CKD	With CKD	p^d^
	N (%)	N (%)^c^	
PCDD/Fs (pg WHO_98_-TEQ_DF_/g lipid)			< 0.001
< 20	1447 (98.4)	24 (1.6)	
≥ 20	1271 (89.1)	156 (10.9)	
Gender			0.044
Men	1463 (94.6)	83 (5.4)	
Women	1255 (92.8)	97 (7.2)	
Age (year)			
< 40	1118 (99.5)	6 (0.5)	< 0.001
40–64	1143 (96.2)	45 (3.8)	
≥ 65	457 (78.0)	129 (22.0)	
Mercury (ppb)			0.743
≤ 20	2519 (93.7)	168 (6.3)	
> 20	199 (94.3)	12 (5.7)	
Metabolic syndrome^a^			< 0.001
No	1891 (95.8)	82 (4.2)	
Yes	695 (88.5)	90 (11.5)	
Diabetes mellitus^b^			< 0.001
No	2355 (95.3)	117 (4.7)	
Yes	362 (85.2)	63 (14.8)	
Hypertension			<0.001
No	2323 (96.4)	88 (3.6)	
Yes	393 (81.0)	92 (19.0)	
Insulin (mU/L)			<0.001
< 22.0	2430 (94.2)	149 (5.8)	
≥ 22.0	162 (87.1)	24 (12.9)	
Uric acid (mg/dL)			< 0.001
≤ 7	2178 (95.7)	98 (4.3)	
> 7	540 (86.8)	82 (13.2)	

^a^defined as meeting three of the following criteria: fasting glucose ≥ 100 mg/dL or under diabetes medication, waist circumference ≥ 90 cm in men or ≥ 80 cm in women, triglycerides ≥ 150 mg/dL or under drug treatment for elevated triglycerides, high-density lipoprotein (HDL) < 40 mg/dL in men or < 50 mg/dL in women, and blood pressures ≥ 130/85 mmHg or under antihypertensive medication; data not available on 140 participants without high dioxin levels.

^b^defined as fasting glucose > 126 mg/dL or under diabetes medication

^c^the prevalence of CKD in participants with the variable

^d^for chi-square test

In the univariate logistic regression analyses, we found a high dioxin level was associated with an OR of 7.40 (95% CI: 4.78–11.45) for CKD in comparison with a lower dioxin level, and other potential risk factors included female gender, MS, DM, hypertension, high insulin, high uric acid, and age above 40 years. (Table 4)

**Table 4:** Risk factors and associated odds ratios for chronic kidney disease among the participants.

Variables	Uni-variate Analyses	Multi-variate Analyses	
	OR (95% CI)^a^	AOR^b^ (95% CI)	AOR^c^ (95% CI)
PCDD/Fs (pg WHO98-TEQDF/g lipid)			
< 20	1	1	1
≥ 20	7.40 (4.78–11.45)*	1.74 (1.02–2.97)*	1.76 (1.04–2.99)*
Gender			
Men	1	1	1
Women	1.36 (1.01–1.84)*	1.74 (1.19–2.54)*	1.74 (1.20–2.53)*
Mercury (ppb)			
≤ 20	1	1	
> 20	0.90 (0.50–1.65)	0.69 (0.34–1.41)	
Metabolic syndrome^d^			
No	1	1	
Yes	2.99 (2.19–4.08)*	1.01 (0.69–1.49)	
Diabetes mellitus^e^			
No	1	1	
Yes	3.50 (2.53–4.85)*	1.24 (0.82–1.87)	
Hypertension			
No	1	1	1
Yes	6.18 (4.53–8.44)*	1.62 (1.11–2.37)*	1.68 (1.17–2.42)*
Insulin (mU/L)			
< 22.0	1	1	1
≥ 22.0	2.42 (1.53–3.83)*	2.04 (1.19–3.49)*	2.14 (1.26–3.61)*
Uric acid (mg/dL)			
≤ 7	1	1	1
> 7	3.38 (2.48–4.59)*	4.38 (2.99–6.40)*	4.25 (2.92–6.20)*
Age (year)			
< 40	1	1	1
40–64	7.34 (3.12–17.26)*	4.70 (1.88–11.73)*	4.66 (1.87–11.62)*
≥ 65	52.60 (23.03–120.11)*	25.67 (10.09–65.32)*	26.66 (10.51–67.62)*

^a^OR: odds ratio; CI: confidence interval

^b^AOR: adjusted odds ratio, the full model included PCDD/Fs, gender, mercury, metabolic syndrome, age, fasting glucose, insulin, and uric acid

^c^AOR:adjusted odds ratio, the final model included PCDD/Fs, insulin, uric acid, and age

^d^defined as meeting three of the following criteria: fasting glucose ≥ 100 mg/dL or under diabetes medication, waist circumference ≥ 90 cm in men or ≥ 80 cm in women, triglycerides > 150 mg/dL or under drug treatment for elevated triglycerides, high-density lipoprotein (HDL) < 40 mg/dL in men or < 50 mg/dL in women, and blood pressures ≥ 130/85 mmHg or under antihypertensive medication; data not available on 140 participants without high dioxin levels

^e^defined as fasting glucose > 126 mg/dL or under diabetes mellitus medication

*p< 0.05 for Wald test

In the multiple logistic regression analyses, the full model showed that a high dioxin level was a risk factor for CKD (AOR = 1.74, 95% CI: 1.02–2.97), independent of gender, mercury level, metabolic syndrome, DM, hypertension, insulin level, uric acid level, and age. (Table 4) However, the regression coefficients associated with the mercury level, metabolic syndrome, and DM did not reach statistical significance. In the final model, a high dioxin level was still associated with CKD (AOR = 1.76, 95% CI: 1.04–2.99), after adjusting for gender, hypertension, insulin level, uric acid level, and age. (Table 4) The variance inflation factor suggests that collinearity among the variables was small. As to the goodness of fit, the final model, which contains seven covariates, had a p value of 0.586 for the Hosmer and Lemeshow test.

## Discussion

In this study, we found an association between a high serum dioxin level (defined as a PCDD/Fs level ≥ 20 pg WHO98-TEQDF/g lipid) and CKD (defined as having an e-GFR ≤ 60 mL/min/1.73m^2^ or diagnosis of CKD by a physician), independent of gender, hypertension, insulin level, uric acid level, and age. This is compatible with the finding in an animal study that the impairment of renal function was related to the toxicity of 2,3,7,8-TCDD [14]. It is generally believed that the renal toxicity of dioxins is mainly introduced through the formation of ROS which is mediated by interacting with the AhR to induce the expression of renal cytochrome P450 (CYP)-A1 and CYP-B1 proteins. [9–11] In fact, ROS might also contribute to the progression of CKD and has become a target of therapeutic intervention. [27] Dioxin might also contribute to CKD through introducing inflammation of kidney tubular cells by activating the AhR and thus inducing Cox-2. [12,28] Tubulointerstitial damage is generally recognized as a common feature of all chronic progressive renal diseases and considered as the final common pathway through which CKD progresses to end-stage renal disease, [29] and inflammation is a critical mechanism that promotes fibrosis and cellular injury within the tubulointerstitium. [30] In addition, because a study found that TCDD might reduce glomerular filtration of rats without affecting the arterial pressure, dioxin might affect the renal function indirectly through extrarenal effects, especially those on the hepatic metabolism. [13] Such an association, however, was not observed in another population with dioxin exposure in Seveso [31]. In that study, level of exposure was estimated using the vicinity of the residence of the participant to the exposure source, instead of measuring personal biological samples as in our study. We speculated that the difference in the accuracy in exposure assessment was a main reason why an association between dioxin exposure and CKD was not observed in that study.

Age is a parameter in the calculation of e-GFR and thus an important risk factor for CKD. A nationwide study in Taiwan found that the prevalence increased with age after 30 years old and doubled between 55–59 years and 60–64 years of age [32], which was compatible with findings in our study. Likewise, a study in the U.S. found that the prevalence of CKD was 3% in participants 40 to 49 years old, 7% in 50 to 59 years old, 14% in 60 to 69 years old, 24% in 70 to 79 years old, and 39% in 80 years and older [33]. In a study in Australia, the prevalence of CKD was 11.2% in participants > 25 years and 54.8% in participants > 65 years [34]. Because dioxin may accumulate in the human body, age also contributes to the blood level of dioxin. After adjusting for age, we found a high dioxin level was an independent predictor of CKD, but the associated AOR was 1.76 in the final model, a substantial decrease from the OR of 7.40 before the adjustment for age, indicating a confounding effect in the univariate analysis.

DM is another risk factor for CKD. A study in the U.S. found that the prevalence of CKD was 13.7% in the population without DM, but was 24.4% in patients with DM [33]. On the other hand, exposure to dioxin has been shown to be related to the development of DM in some previous studies. A study of servicemen retired from the Vietnam War found increased fasting blood sugar when the 2,3,7,8-TCDD serum level was higher than 94 pg WHO98-TEQDF/g lipid [35]. Another study compared Vietnam War retired serviceman with a mean serum 2,3,7,8-TCDD level of 12.2 ppt to a control group with a mean level of 4.0 ppt found them at higher risks of having fasting blood glucose abnormality, high insulin levels, and DM [36]. In a study of weed killer user, the serum level of 2,3,7,8-TCDD was higher than 1500 pg/g lipid, and the prevalence of DM was as high as 60% [37]. In Belgium, a study found that DM patients’ serum dioxin levels (including PCDD/Fs and four kinds of PCBs) were 1.62 times higher than those of who did not have DM and that a serum PCDD/Fs in the top 10% was associated with an OR of 5.1 for having DM [38]. Because DM is a common cause of renal function impairment, it was another potential confounder in our study. After adjusting for other risk factors including DM (AOR = 1.24, 95%CI: 0.82–1.87) and high insulin level (AOR = 2.04, 95%CI: 1.19–3.49) in the full model of multiple regression analyses, however, we still found that a high dioxin level was an independent predictor of CKD (AOR = 1.74, 95%CI: 1.02–2.97). Therefore, dioxin might cause CKD through mechanisms other than causing DM first and then leading to CKD.

Likewise, hypertension is another risk factor for CKD, being the second leading cause of CKD in the U.S. and noted in 80%-85% of CKD patients [39]. A study demonstrated that hypertension could be induced by treating animal with TCDD [40]. In humans, a study found that the prevalence of hypertension varied inversely with GFR, increased from 66% at a GFR of 83 ml/min/1.73m^2^ to 95% at a GFR of 12 ml/min/1.73m^2^ [41]. In Taiwan, a study found high levels of PCBs and PCDFs were associated with hypertension in women (AOR = 3.5, 95% CI: 1.7–7.2) [42]. A previous study of our team also found that increased serum PCDD/F levels were associated with increased diastolic blood pressure [25]. In an animal model, chronic exposure of TCDD was found to sustain AhR activation, leading to systemic hypertension and left ventricular hypertrophy, which might be mediated in part by increased superoxide [43]. Hypertension is a common cause of CKD, and therefore it was an important potential confounder in our study. After adjusting for other risk factors including hypertension in the multiple regression analyses, however, we still found that high dioxin was an independent risk factor of CKD. Therefore, in addition to causing hypertension that can in turn lead to CKD, dioxin might cause CKD through other mechanisms.

In the Yuso study, hyperuricemia was found to be associated with high dioxin levels [44]. Another study of incinerator workers with chronic exposure to PCDDs, PCDFs and coplanar PCBs also had high serum uric acid levels [45]. In a previous study of our team, we found higher serum dioxin levels were associated with higher uric acid levels (AOR = 2.20 for 25th to 50th percentile [95%CI: 1.30–3.73]; AOR = 1.86 for 50th to 75th percentile [95%CI = 1.08–3.22]; and AOR = 3.00 for ≥ 75th percentile [95%CI: 1.69–5.31]) [46]. The relationship between uric acid and CKD is very complicated because it had many potential confounding variables such as hypertension, DM, and MS [47]. The possible mechanisms include induction of afferent arteriopathy, inflammation, and activation of the renin-angiotensin system [48]. Our study showed that uric acid was an independent risk factor of CKD after adjusting for gender, age, hypertension, and insulin level.

There were some limitations in our study. We did not have data on the serum PCDD/Fs level over time and the measurements had ended about three years before the interview and health examination ended. However, because the half-life of PCDD/Fs in the serum is as long as 7 years [49] and the sources of the environment contamination remained similar during that period, we believe the level remained similar in a given participant over the years. In fact, most of the previous studies on the health effects of dioxin also had only one measurement over the study period. Even though the measurements of PCDD/Fs were made three years before the assessment of CKD, it is possible that the onset of CKD had occurred before the measurement, and this reduced the strength of the association serving as an evidence of a causal relationship. In addition, we collected data on medical history through self-administrated questionnaires, but we were unable to confirm them because this is a community-based study. Furthermore, strictly speaking, in order to make the diagnosis of CKD, the renal function should be followed for at least 3 months, but we did not do follow-up measurements in this study. While this study cannot provide strong support to a causal relationship between the exposure and CKD due to these limitations, we have discussed evidence showing that such a causal relationship is biologically plausible.

The environmental pollution observed in our study was not unique. In China, the Ya-Er Lake also received a large amount of waste water from a nearby chloroalkali plant and was heavily polluted by PCDD/Fs, and a study found high levels of PCDD/Fs in surface sediment, soil, human hair, and fish muscle [50]. On the other hand, a study compared the PCDD/Fs levels of surface soil collected in the vicinity of a large scale e-waste recycling facility in Taizhou, China to those of surface soils from a chemical industrial complex (a coke-oven plant, a coal-fired power plant, and a chloroalkali plant) in Shanghai and found that the total PCDD/Fs levels in samples from the chemical industrial complex were lower [51]. Therefore, as the e-waste recycling industry in China becomes the largest in the world [52], environmental PCDD/Fs exposure becomes a serious threat to the public health. In fact, a review found that other sources of environmental PCDD/Fs exposure included municipal waste incineration, mineral fuel usage, open burning crop residues, discharge of industrial wastes, and vehicle exhaust emission [53]. Altogether, environmental PCDD/Fs exposure is an emerging health problem in world. While there are some animal studies supporting a possible association between dioxin exposure and CKD, using “dioxin” and “chronic kidney disease” to search in the PubMed, we failed to find any previous epidemiological studies focusing on this association. Therefore, the present study is most likely to be the first epidemiological study on this issue.

## Conclusion

In an endemic area of dioxin exposure in Taiwan, we observed an association between high blood dioxin levels and CKD. Whereas older age, female gender, hypertension, high insulin, and high uric acid were also found to be risk factors, a high dioxin level was an independent predictor of CKD after adjusting for the effects of those factors. Whereas the mechanisms by which dioxin causes renal toxicity are still unclear and need further research, results of this study suggested that there might be pathways other than the association between dioxin and hypertension or DM. As dioxin becomes an emerging environmental health hazard globally, the kidney function of exposed populations should be monitored.

## Supporting Information

S1 Table The data for tables. (XLSX)
